# Constant-pH Molecular Dynamics of Cationic Peptide Dendrimers Binding to siRNA

**DOI:** 10.1021/acs.jcim.5c02636

**Published:** 2026-02-05

**Authors:** Filipe E. P. Rodrigues, Tamis Darbre, Miguel Machuqueiro

**Affiliations:** † BioISI − Instituto de Biossistemas e Ciências Integrativas, Departamento de Química e Bioquímica, Faculdade de Ciências, 111161 Universidade de Lisboa, 1749-016 Lisboa, Portugal; ‡ Department of Chemistry, Biochemistry and Pharmaceutical Sciences, University of Bern, Bern CH-3012, Switzerland

## Abstract

Transfection, the process of delivering genetic material into eukaryotic cells, is crucial in biotechnology and the development of treatments. Naked nucleic acids face challenges such as enzymatic degradation, poor pharmacokinetics, and immunogenicity, which can be mitigated by delivery systems such as liposomes, cationic polymers, and dendrimers that protect and enhance uptake. Peptide dendrimers, in particular, show promise as nucleic acid carriers due to their lower cytotoxicity and immunogenicity, though their mechanisms, efficiency, and optimization remain to be clarified. Here, we characterized the configurational, conformational, and protonation landscapes of different peptide dendrimers in complex with siRNA. We found that nucleic acids modulate dendrimer structure, with electrostatic interactions strengthened at low pH through enhanced protonation of the N-termini. Although experimental data show that the more hydrophobic dendrimer examined displays the highest apparent affinity for siRNA, its reduced number of lysine residues results in weaker overall binding due to diminished charge density. This higher affinity observed is likely linked to increased aggregation propensity. In contrast, the dendrimer sequence with branching residues of inverted chirality, which performs worse, shows the lowest propensity for aggregation. Our work suggests that chirality has only a negligible effect on the dendrimer–siRNA binding modes, and that such differences are subtle, particularly at the monomeric level. Overall, this work provides mechanistic insight into dendrimer–siRNA interactions and outlines potential strategies to refine dendrimer design for improved nucleic acid delivery.

## Graphical abstract

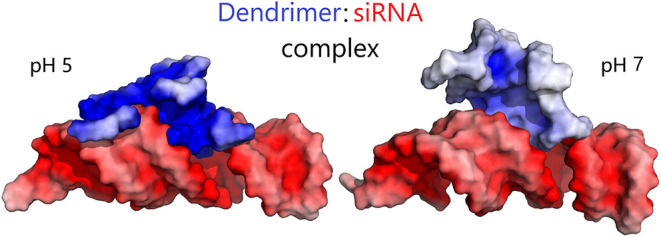

## Introduction

Transfection is the process of introducing exogenous genetic material into a eukaryotic cell, and it is vital for several applications, ranging from biotechnology to therapeutics.^1,2^ The latter applications use transfection to study gene expression and for siRNA therapies. siRNA-mediated gene repression is based on Watson–Crick base pairing with an mRNA molecule. This highly specific interaction promotes the degradation of the target mRNA by the RNA-induced silencing complex, leading to gene silencing.^3,4^ Naked siRNA, however, is susceptible to endonucleases and exonucleases, has poor pharmacokinetic properties, and can trigger an immunogenic response.^2,5^ To avoid these undesirable effects, siRNA molecules can be coupled with a delivery system that protects their cargo while delivering it to target cells.^5^

Drug delivery systems can range from chemical modifications of siRNA^6^ to encapsulating or conjugating siRNA molecules with liposomes, cationic polymers such as polyethylenimine and polypropyleneimine, or cationic dendrimers, including polyamidoamine and peptide dendrimers.^2,5,7^ Liposomes are the most used nonviral vectors and have been added to a clinically approved drug using a formulation of liposomes carrying a chemically modified siRNA (ONPATTRO).^8,9^ However, there are still risks associated with the use of cationic liposomes, such as the possibility that these structures may promote the formation of reactive oxygen species, inducing cytotoxicity.^10^

PAMAM (polyamidoamine) and PPI (polypropyleneimine) dendrimers have been extensively studied for several medical applications, including gene transfection.^11^ More recently, peptide dendrimers entirely composed of amino acid residues have been reported to interact with various biological targets, resulting in strong efficacy as antimicrobial agents,^12,13^ pathogenic biofilm inhibitors,^12,13^ drug delivery systems,^14^ and efficient vectors for DNA, siRNA and small oligonucleotides.^15–20^ The efficiency is primarily due to the presence of cationic amino acid residues, such as lysine and arginine, which enhance the binding affinity to the negatively charged nucleic acids. The positive charges also promote membrane interactions, whether slightly anionic or bearing anionic groups at the surface, facilitating adhesion to target cells.^21^ This enhanced adsorption to the cell membrane may also facilitate endosomal-mediated internalization. This process can be further improved by functionalizing the peptide dendrimers with lipophilic tails, acting as anchors to the cellular membrane.^15,16^ However, achieving a balance among the transfection efficiency of the genetic material, cytotoxicity, and immunogenicity remains challenging.^22^ Hence, the choices regarding the dendrimer sequence and electrostatic properties are paramount for the effectiveness of these systems.

Recently, two promising peptide dendrimer sequences, MH18 and MH13, have been discovered.^16–18,21^ These third-generation dendrimers are constituted by leucines and lysines, with MH18 featuring a tetra-leucine hydrophobic core and MH13 possessing two palmitoyl groups as its core. These dendrimers do not require additional cationic lipids for activity, in contrast to the previously described peptide dendrimers without hydrophobic cores.^19,20^ However, it was observed that altering the chirality or introducing mutations in the MH18 or MH13 sequences enabled researchers to modulate nucleic acid affinity and overall transfection performance.^16^ The mutated dendrimer MH47, in which the four lysines in G2 were mutated to leucines, resulted in tight nucleic acid binding but poor transfection efficiency. Another sequence, MH18D3, had the chirality of its branching lysines switched from l- to d-amino acids, resulting in a weaker RNA binding capability and, consequently, poor transfection efficacy. Additionally, the enantiomeric d-sequences DMH18 and DMH13 showed activity comparable to that of their l-sequence counterparts. The significantly decreased activity of stereoisomer MH18D3 or mutated MH47 could not be explained by the experimental results at hand. Due to their branched topology, dendrimers can adopt more rigid and globular structures.^23^ However, the precise structure of dendrimers in solution remains poorly defined, mainly because they can adopt a large number of conformations despite their topological constraints.^23^

In our previous work, we explored the conformational space of peptide dendrimers both in the aqueous phase and in interaction with a membrane model.^24^ We observed that their overall properties were mainly influenced by environmental pH, with higher pH resulting in a smaller size. The only significant distinction observed among the sequences was that MH47, compared with the other dendrimer sequences, is more compact at lower pH.^24^ Despite these findings and the important experimental evidence available, significant molecular-level knowledge remains to be uncovered regarding the interactions between cationic peptide dendrimers and nucleic acids. For example, the reasons why different combinations of l-to-d substitutions in MH18 residues result in varying effects on siRNA affinity, transfection efficiency, and nucleic acid release^16^ remain poorly understood. Computational methods have proven invaluable for studying these processes, as they provide molecular details that are difficult to obtain, if not impossible, with most experimental methods.

In this work, we present a detailed characterization of the structure and protonation changes of the dendrimers MH18, DMH18, MH18D3, and MH47 upon interaction with siRNA. We further evaluate how endosomal acidification (pH 5.0) influences binding modes and affinities, which we estimate using both pH-dependent MM/PBSA calculations and the Wyman–Tanford linkage framework. These results were also used to interpret and combine the available experimental data for these dendrimers, which will be essential for the future design of new dendrimers with enhanced transfection capabilities.

## Methods

### System Setup and MM/MD Settings

All simulations were performed with the Amber ff14SB^25,26^ and the GROMACS 2021.2 ^27^ package. Since there are no structures for the siRNA-dendrimer complex, we need to develop an MD-based docking protocol to obtain starting configurations for the complex. Hence, we built models for the individual components of the complex, the dendrimer and siRNA (PDB: 5N8L; 5′-UUAAUUAUCUAUUCCGUACUU-3′) molecules. We used PyMOL^28^ to build the initial conformations of the dendrimers. The free lysine side chains were constructed in their deprotonated form, while the free N-termini were built in their protonated form. The C-terminus was capped with an NH_2_ group. The RNA bases and phosphate groups were not included in the list of titratable sites. This is an acceptable approximation, since these groups, even at the lowest pH value used (5.0), should protonate only sparingly.^29^ These structures were then submitted to an energy minimization protocol and subsequent initialization procedure before production simulations. The energy minimization consisted of two steps, each with 10,000 integrator steps. Both steps used the steepest descent algorithm. The first left all bonds unconstrained, and the second used p-LINCS^30^ and SETTLE^31^ constraining algorithms applied to the solute and solvent, respectively, for all hydrogen-containing bonds.

The initialization protocol consisted of 200 ps of NVT MD with the v-rescale thermostat^32^ set for a reference temperature of 310 K, with a temperature coupling of 0.01 ps. Starting velocities were assigned from a Maxwell distribution representative of 310 K. This was followed by 200 ps of NPT MD with the Parrinello–Rahman isotropic barostat^33^ set for a reference pressure of 1 bar and pressure coupling of 1 ps (temperature coupling was also increased to 0.5 ps). Both of these steps used an integration step of 1 fs. Long-range electrostatics were treated with the Particle Mesh Ewald (PME) method,^34^ with a Verlet cutoff scheme of 1.0 nm, a Fourier space grid of 0.12 nm, an interpolation order of 4, and the neighbor list was updated every 10 steps. van der Waals interactions were truncated at 1.0 nm. The integration step was updated to 2 fs in all subsequent MD steps. After this initialization step, 10 replicates of 150 ns production CpHMD simulations, at pH 5.0 and 7.0, were performed.

Since long-range electrostatics are treated with PME,^34^ simulations require charge neutrality. An initial estimate of the number of counterions was obtained from previously performed simulations of these systems.^24^ After a 10 ns segment of CpHMD is performed, the system’s average charge is assessed to determine whether it deviates from system neutrality. This approach involves iterative steps of performing short CpHMD segments to evaluate and adjust the number of counterions, thereby maintaining the system near charge neutrality. Therefore, we solvated our system in a rhombic dodecahedral box containing 14,000–16,000 water molecules, using the tip3p^35,36^ water model, applying periodic boundary conditions, and adding the appropriate number of counterions for each system and pH value (Table 1).

**Table 1:** Summary Table of All Dendrimer Sequences Simulated^a^ ^16^

system	sequence	free siRNA (%)	pH	# water	# ions
MH18	(KL)_8_(*K*KL)_4_(*K*LL)_2_ *K*LLLL	4.8 ± 0.4	5	14,002	48 K; 28 Cl
			7		50 K; 26 Cl
DMH18	(kl)_8_(*k*kl)_4_(*k*ll)_2_ *k*llll	4.6 ± 0.7	5	14,412	48 K; 28 Cl
			7		50 K; 26 Cl
MH18D3	(KL)_8_(*k*KL)_4_(*k*LL)_2_ *k*LLLL	18.8 ± 3	5	16,371	54 K; 34 Cl
			7		56 K; 32 Cl
MH47	(KL)_8_(*K*LL)_4_(*K*LL)_2_ *K*LLLL	3.0 ± 0.1	5	14,218	50 K; 26 Cl
			7		52 K; 24 Cl

a A one-letter code for amino acids was used, where uppercase letters represent l-amino acids, and lowercase letters represent d-amino acids. Italic letters represent branched lysines. All Free siRNA experimental data were obtained from ref 16.

After obtaining starting conformations for both the dendrimer and the siRNA, simulations of the complex were performed. The dendrimers were first placed ∼3 nm away from the siRNA and simulated using MD to sample their nucleic acid approach pathways unbiasedly. The simulations were stopped when the dendrimer reached the nonbonded interactions cutoff distance (∼1.0 nm) from the siRNA. We then removed the extra water molecules from the system in the last configurations, leaving a minimum distance of ∼1.6 nm to the periodic images. Since the CpHMD simulations are computationally intensive, this system-reduction step was essential to improve their performance. The final system configurations were then subject to an additional set of minimization/initialization protocols (see above).

### Poisson–Boltzmann/Monte Carlo and CpHMD Settings

We performed production simulations with our implementation of the stochastic CpHMD method,^24,26,37–40^ which couples the conformational sampling of MD simulations with the protonation state sampling provided by Poisson–Boltzmann/Monte Carlo (PB/MC) calculations. In this method, a CpHMD cycle begins with a PB/MC step, in which we calculate the free energies of each protonation (tautomer) state for our titrating groups using the DelPhi V5.1 program.^41^ We use a dielectric constant of 2 ^42^ for the solute. At the same time, the solvent is treated implicitly and with a high dielectric constant of 80.^42,43^ The molecular surface of the solute was defined with a probe with a radius of 1.4 Å. The populations of each tautomer within each protonation state are sampled using PETIT v1.6.1,^44^ which employs an MC scheme based on free energy terms calculated in the previous PB step. A total of 10^5^ MC cycles were performed for each conformation, with the protonations of the last cycle selected as the new protonation states. The next step in the CpHMD cycle is a short solvent-relaxation MD step of 0.2 ps, with the solute frozen, to allow solvent molecules to adjust to the new protonation states. The cycle is then completed with a 20 ps production MD segment, which samples the system’s conformational space with the new solute protonation states. This procedure is then repeated iteratively.

### Analyses

All analyses were performed using the GROMACS 2021.2 software package and in-house tools. The protonation of each titrating group was analyzed individually.

#### Position along the siRNA Double Strand

The position of the dendrimer relative to the siRNA double strand provides a measure of the dendrimer’s location along the siRNA molecule’s length. This property was calculated by determining the distance between the geometric center of the dendrimer and the closest siRNA end. In addition, we calculate the angle formed between the geometric center of the dendrimer and the geometric centers of each end of the siRNA. The cosine of this angle determines the distance between the closest end of the siRNA and the projection of the dendrimer on the siRNA molecule. This distance can be converted to a percentage relative to the length of the siRNA molecule (Figure 1).

**Figure 1:**
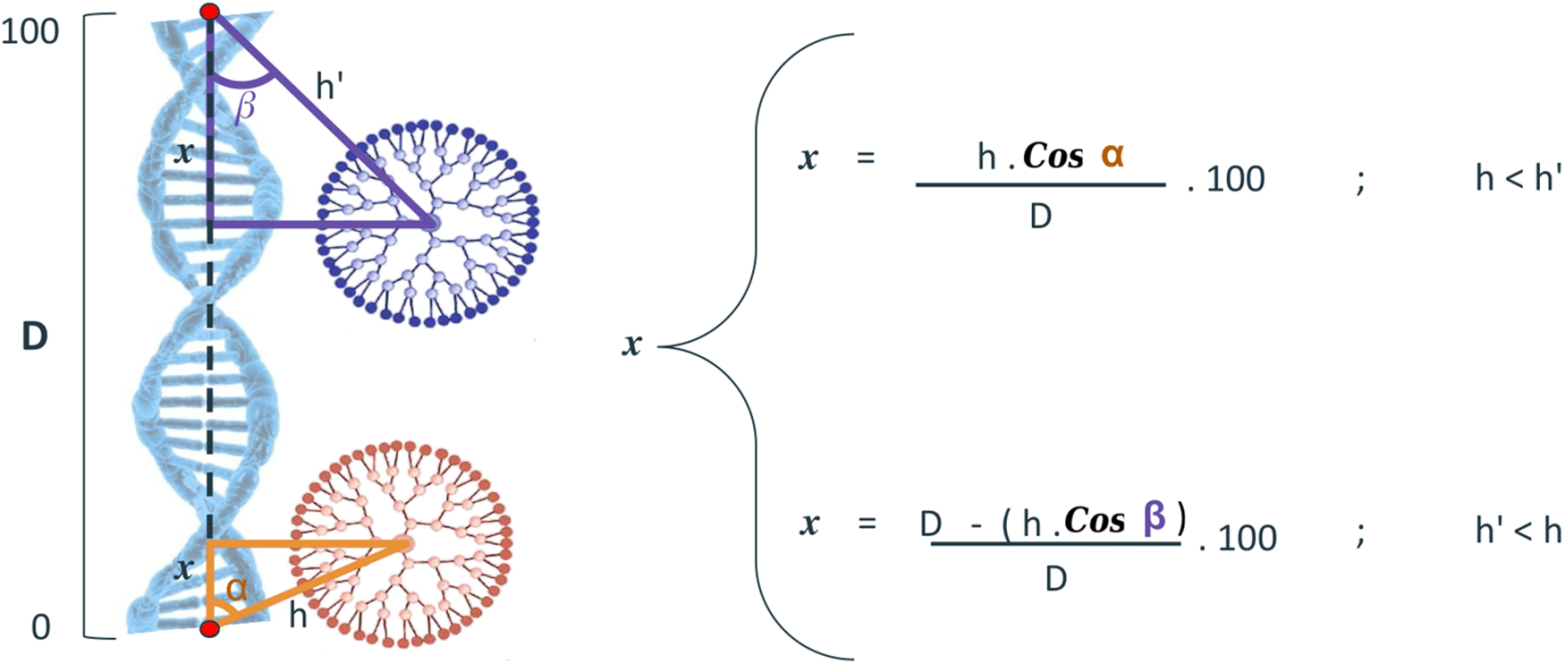
Scheme illustrating the calculation of the dendrimer positioning on the siRNA length vector. The calculation was normalized to a range of 0 to 100. To this metric, there is no distinction between the two RNA strands.

#### Bending of the siRNA Double Helix

The bending of the siRNA double helix was calculated as the angle formed by three points, one on each end of the siRNA molecule, and one on its center. To minimize artifacts in this measurement, as the points are not centered on the siRNA molecule, we defined them as the center of geometry of the selected nucleotide pairs. On both extremities of the siRNA, the outermost paired nucleotides were used, while for the central point, the central 7-nucleotide pairs were used.

#### Electrostatic Shielding of siRNA

The electrostatic shielding of the dendrimer on the siRNA phosphate groups provides a measure of the coverage of the negatively charged phosphates in the nucleic acid by the dendrimer. This was determined by calculating the percentage of solvent-accessible surface area (SASA) of the phosphate groups that is covered by the dendrimer. By calculating the SASA of these phosphate groups in the presence and absence of the siRNA molecule, we obtain the fraction (percentage) of coverage, which we refer to as shielding.

#### Major/minor Groove Mapping

The mapping of the dendrimer on the surface of the siRNA, namely whether a given titrable group is more internalized in the major groove, more exposed to the minor groove, closer to a phosphate group, or more solvent exposed, was based on distance criteria (Figure 2). This distance criterion consisted of calculating the minimum distance between each titrating nitrogen atom and the two sets of atoms located in the minor or major grooves. The shortest distance assigns that nitrogen atom to either the major or the minor groove.

**Figure 2:**
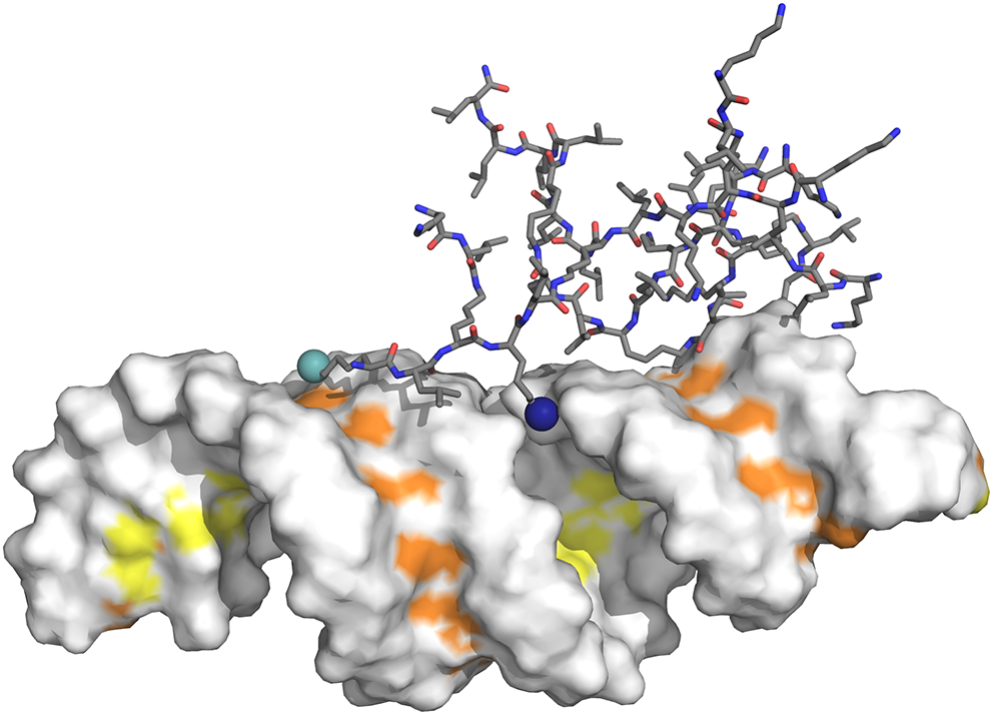
Scheme illustrating how we calculate the dendrimer mapping on the siRNA. The dendrimer is represented as sticks with carbon atoms in gray. Hydrogen atoms were omitted for clarity in the image. The two spheres correspond to lysine side-chain nitrogen atoms located closer to the minor and major grooves, colored light blue and dark blue, respectively. The siRNA is represented with a white surface with two sets of atoms, corresponding to the minor and major grooves, colored in orange and yellow, respectively.

#### Energy Landscapes

The conditional free energy landscapes were calculated from a probability density function of a two-dimensional space, using the distance to the closest siRNA groove and the distance to the closest phosphate group as coordinates. The probability density functions were estimated using a Gaussian kernel estimator with a grid spacing of 0.009 Å^3^.^45^ The conditional energy (*E*_(*r*)_) surfaces were computed with the following (eq 1)­

1 $$E_{\left(r\right)}=-\mathrm{RT}\frac{\ln P_{\left(r\right)}}{P_{\max}}$$

where *R* and *T* are the ideal gas constant and temperature, respectively, while *P*_(*r*)_ and *P*_*max*_ are the probability density function and its respective maximum.

#### pH-Weighted MM/PBSA

The binding free energy was calculated using PyBindE,^46,47^ an implementation of the Molecular Mechanics Poisson–Boltzmann Surface Area (MMPBSA) method. This approach uses a single-trajectory protocol, in which entropic contributions are reduced by the cancellation of conformational fluctuations, making it suitable for comparing different environmental conditions while maintaining an essentially unchanged protein structure. The same conformational ensemble is employed for the complex, protein, and ligand, and the binding free energy (Δ*G*_bind_) is defined as Δ*G*_bind_ = Δ*G*_complex_ – (Δ*G*_protein_ + Δ*G*_ligand_). Within a thermodynamic cycle, Δ*G*_bind_ is decomposed into vacuum (*E*_VdW_ and *E*_coul_) and solvation (*Solv*_polar_ and *Solv*_apolar_) contributions. The polar solvation energies were computed using DelPhi4Py ([[https://github.com/mms-fcul/DelPhi4Py]­(https://github.com/mms-fcul/DelPhi4Py)]), with dielectric constants of 4 and 80 used for the solute and the solvent, respectively. Several studies have shown that using a dielectric constant in the range of 2–8 improves the prediction of experimental data.^48–50^ A grid scaling factor of 2 and a convergence criterion of 0.001 were used, with a calculation rate of 100 ps. The calculation provides separately four different contributions to the final *E*_*bind*_, namely *E*_*Coul*_, *E*_*VdW*_, *Solv*_*polar*_, and *Solv*_*apolar*_. The obtained energy values are correctly weighted by the simulation pH, as PyBindE utilizes the correct protonation states obtained from CpHMD simulations.

#### Wyman–Tanford Linkage

Methods such as MMPBSA and MMGBSA can be used to estimate binding energies based on force field parameters and Poisson–Boltzmann or generalized-Born calculations, respectively. However, these are reliant on the adequacy of the system’s parameters and the force field used in the simulations. Hence, when calculating differences in Δ*G* across different pH values, the resulting ΔΔ*G* can be influenced by these factors, making it harder to detect significant differences. This ΔΔ*G* can be more directly obtained through a direct (and exact) relation between free energy and the total charge difference between different pH values. This relation is obtained through the Wyman–Tanford linkage formalism,^47,51–53^ which establishes the following thermodynamic relation (eq 2)­

2 $$\Delta \Delta G=2.303\mathrm{RT}\left(\Delta Q_{\mathrm{pH}}-\Delta Q_{pH^{\mathrm{ref}}}\right)$$

where ΔΔ*G* is the free energy difference relative to a pH variation, *R* is the perfect gas constant, *T* is the temperature, in Kelvin, of the system, and the Δ*Q*_*pH*_ and Δ*Q*_*pH* ^*ref*^_ are the charge differences between bound and unbound states, at pH 5 and 7, respectively. In this case, pH 7 was chosen as the reference value to measure the energetic gain upon transitioning to a more acidic environment.

#### Protonation Analysis

The protonation of each titrating group, namely the N-termini (G3-NTR), third-generation lysine side chains (G3-side), and the second-generation lysine side chains (G2), was analyzed as a whole with all sites in each group treated as replicates, taking advantage of the pseudosymmetry of the dendrimers.

## Results and Discussion

### Equilibration

We performed CpHMD simulations of MH18, MH18D3, DMH18, and MH47 in a complex with a siRNA molecule. To assess the equilibration and convergence of these systems, as well as the stability of the formed complexes, we analyzed several properties. We calculated the radius of gyration, the charge of the dendrimer, the RMSD, and the siRNA bending angle to evaluate the convergence of these properties (Figure S1 of Supporting Information). These properties provide insights into the effects of dendrimers on siRNA stability and the impact of the nucleic acid on the structure and charge of the dendrimers.

Regarding the radius of gyration and the charge of the dendrimers, these properties exhibit quite different behavior. While the charge of the dendrimers remains stable during our simulations (owing to fast convergence), the radius of gyration exhibits slower equilibration. In addition to the heterogeneity observed for this property, its values also significantly deviate from those observed in the unbound state. Notwithstanding, MH47 is the dendrimer sequence that most closely resembles the radius of gyration values observed in solution. This is likely due to the lower number of charges on this dendrimer sequence, which results in lower forces exerted by the negatively charged siRNA and less deformation of the dendrimer’s structure than in water. Overall, these two properties provide insight into the effect of the siRNA on the dendrimer, namely that it partially distorts the globular structure of the dendrimer, making it dependent on the proximity to the siRNA, while overall making the dendrimers more charged than in the aqueous phase^24^ (2nd row in Figure S1 of Supporting Information). The RMSD and siRNA bending angle show that the nucleic acid is not affected by the presence of the dendrimer. Excluding two replicates at pH 7 of DMH18, the siRNA does not significantly bend below 160°. This, accompanied by low RMSD values (below 0.6 Å), indicates that the siRNA molecule does not deform significantly in the presence of the peptide dendrimer.

After assessing the impact each interacting partner has on the other, we can also evaluate the overall stability of the complex. We calculated the position of the dendrimer along the siRNA double-helix vector, the contact interface area of the complex, the number of electrostatic contacts between dendrimer amines and siRNA phosphates, and the overall electrostatic shielding in the siRNA phosphates provided by the dendrimer (Figure S2 of Supporting Information). The interfacial area is well-converged, with only small deviations in one replicate of DMH18 (Figure S3 of Supporting Information). The number of phosphate interactions and the resulting shielding property show larger fluctuations across replicates, whereas all fluctuate within the same range. The position of the dendrimer along the siRNA length vector shows the most remarkable heterogeneity, with dendrimers docking to the siRNA surface and forming a stable interface at random locations. However, considering the periodic nature of the nucleic acid structure, the dendrimer:siRNA binding modes may be significantly more homogeneous. Interestingly, there are a few replicates across almost all systems in which the dendrimer slides and interacts with the siRNA 3′ and 5′ ends (positions <0 and >100).

Overall, we observe that the dendrimer does not significantly perturb the siRNA; however, the presence of the nucleic acid does modulate its structure. Therefore, all structural properties were considered converged only after the initial 50 ns of our simulations, which were discarded. In some cases, the replicate data remained heterogeneous, even though they fluctuated within the same range. Indeed, for some processes, the system equilibration may occur on a time scale inaccessible to our CpHMD simulations, so we mitigated this by using 10 replicates.

All dendrimers show a significant difference in charge between pH 5 and 7 (Figure S1 of Supporting Information). However, for all analyzed structural properties, no differences were observed between the two pH values, with only small trends in some dendrimer sequences. This observation suggests that nucleic acid presence can shield the total charge difference, making many structural properties more homogeneous. All siRNA:dendrimer complexes with identical interfacial areas and electrostatic contacts may indicate similar binding energies. Given that available experimental results suggest different affinities for nucleic acids, we will further investigate the conformational, configurational, and protonation landscapes of these complexes to aid in interpreting the experimental data.

### The Dendrimer:Nucleic Acid Interface

Although some properties like the dendrimer charge, radius of gyration, the siRNA bending, and its RMSD, were mainly used to follow the equilibration of the dendrimer docking process to the nucleic acid, other properties, including the interfacial area, number of phosphate-amine contacts, and the resulting electrostatic shielding, can provide helpful information to compare between dendrimers (Figure 3). The interfacial area remained relatively constant across different dendrimer sequences and pH values. This suggests that the shared topology of dendrimers affects their adaptation to the siRNA surface more than simple electrostatics alone. Upon closer examination of the electrostatic shielding (i.e., the percentage of phosphate surface covered by the dendrimer), we observe that MH47 is less efficient at covering the siRNA phosphate groups at pH 7. The lower overall charge of this dendrimer limits the number of electrostatic interactions with the nucleic acid at pH 7. Since this dendrimer still covers the same area of nucleic acid, the lower number of phosphate interactions indicates that there is an optimal number of positive charges complementary to the phosphate groups, which MH47 does not satisfy at neutral pH. Instead, this dendrimer features a higher number of hydrophobic (Leu) residues in the siRNA interaction, especially at pH 7 (Figure S4 of Supporting Information). This is to be expected since MH47 is more hydrophobic than the remaining dendrimers, regardless of pH (Figure S5 of Supporting Information).

**Figure 3:**
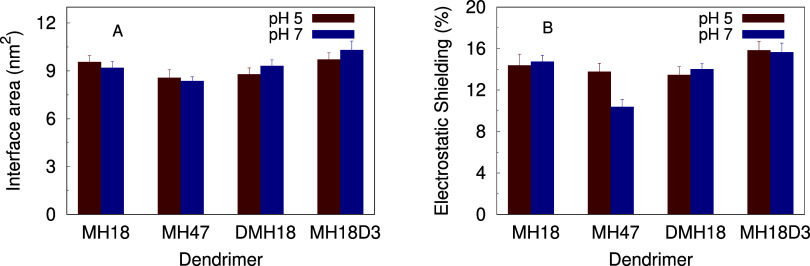
Interfacial area of the dendrimer:siRNA complexes (A) and the percentage of electrostatic shielding (B), for each system, over pH.

### The pH Titration of Peptide Dendrimers

We calculated the titration curves of the N-termini amino groups for all dendrimers in their apo and siRNA-bound forms (Figure 4). Data for lysine side chains are not shown because they remain mostly protonated over the simulated pH range. From the N-termini pH titration data, we can fit Hill equations and obtain average p*K*_a_ values (Table 2). These “apparent” p*K*_a_ values provide an estimation of the proton binding affinities of the N-termini, as a whole, and can be interpreted as a sensor of the local electrostatics. In fact, since there are 8 N-terminal sites, we are measuring an average effect. As an example, we observe a slightly higher p*K*_a_^*apo*^ for the N-termini of MH47 (Table 2), which is justified by its lower overall positive charge, which allows these amino groups to start to protonate at higher pH values.

**Table 2:** List N-termini p*K*_a_ Values Calculated for All Dendrimers in Water and Bound to siRNA^a^

Sys	p*K*_a_^*apo*^	*n*	p*K*_a_^*bound*^	*n*	p*K*_a_^*phos*^	p*K*_a_^*groove*^
MH18	6.4 ± 0.0	1.0 ± 0.0	7.2 ± 0.1	0.7 ± 0.0	7.7 ± 0.2	7.2 ± 0.1
MH18D3	6.2 ± 0.0	0.8 ± 0.0	7.2 ± 0.1	0.5 ± 0.1	7.8 ± 0.2	6.7 ± 0.1
DMH18	6.3 ± 0.0	1.0 ± 0.0	7.1 ± 0.1	0.7 ± 0.0	7.8 ± 0.2	6.9 ± 0.1
MH47	6.6 ± 0.0	0.9 ± 0.0	7.1 ± 0.1	0.6 ± 0.0	7.8 ± 0.2	6.9 ± 0.1

a The *phos* and *groove* labels correspond to the N-termini amino groups interacting with the siRNA phosphate groups (within 0.4 nm) or either of its grooves (within 0.6 nm), respectively.

**Figure 4:**
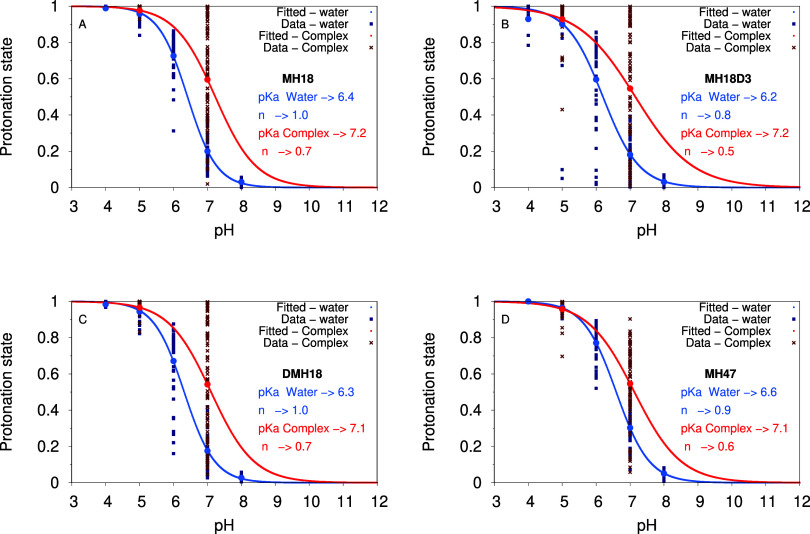
Dendrimer N-termini protonation over pH for all dendrimers in the apo (blue) and siRNA-bound (red) forms. Hill curves were fitted to the data and shown as solid lines. All 80 data points are shown (10 replicates × 8 N-termini). The global average protonations are also shown as solid circles.

In the siRNA-bound systems, there is a decrease in the Hill coefficient values that can be attributed to strong coupling between the N-termini, leading to cooperativity in the proton binding process. Furthermore, an almost systematic increase in the p*K*_a_ values can be also observed upon binding to siRNA compared to the dendrimers in solution (Table 2). This can be explained by the interaction of these groups with the negative phosphate groups of the siRNA, which stabilizes their protonated form. This effect clearly surpasses the also expected desolvation effect, which would lead to a decrease in p*K*_a_. This resulting positive shift indicates that electrostatics play a dominant role in these binding phenomena. To quantify this effect, we calculated the p*K*_a_ values for the N-termini when interacting with the siRNA molecule near the major/minor grooves or the phosphate groups (Table 2). Regardless of whether the interaction is with a groove or a phosphate group, we always observe an increase in the p*K*_a_ values relative to the apo form, confirming that electrostatics prevails over desolvation effects. Although this effect is maximized when the N-termini interact directly with the phosphate groups, it still holds when the N-termini insert into the more solvent-shielded grooves, since these regions also experience a very negative electrostatic potential. These positive p*K*_a_ shifts lead to an overall increase in the peptide dendrimers’ total positive charge.

To expand and obtain a visual analysis of the impact of siRNA groups (phosphate vs grooves) in the conformational and protonation space of the dendrimers, we have mapped the relative position of the titrable groups when they interact with the nucleic acid, and observe how their protonation change in response to their location (Figure 5 and Table S1 of Supporting Information).

**Figure 5:**
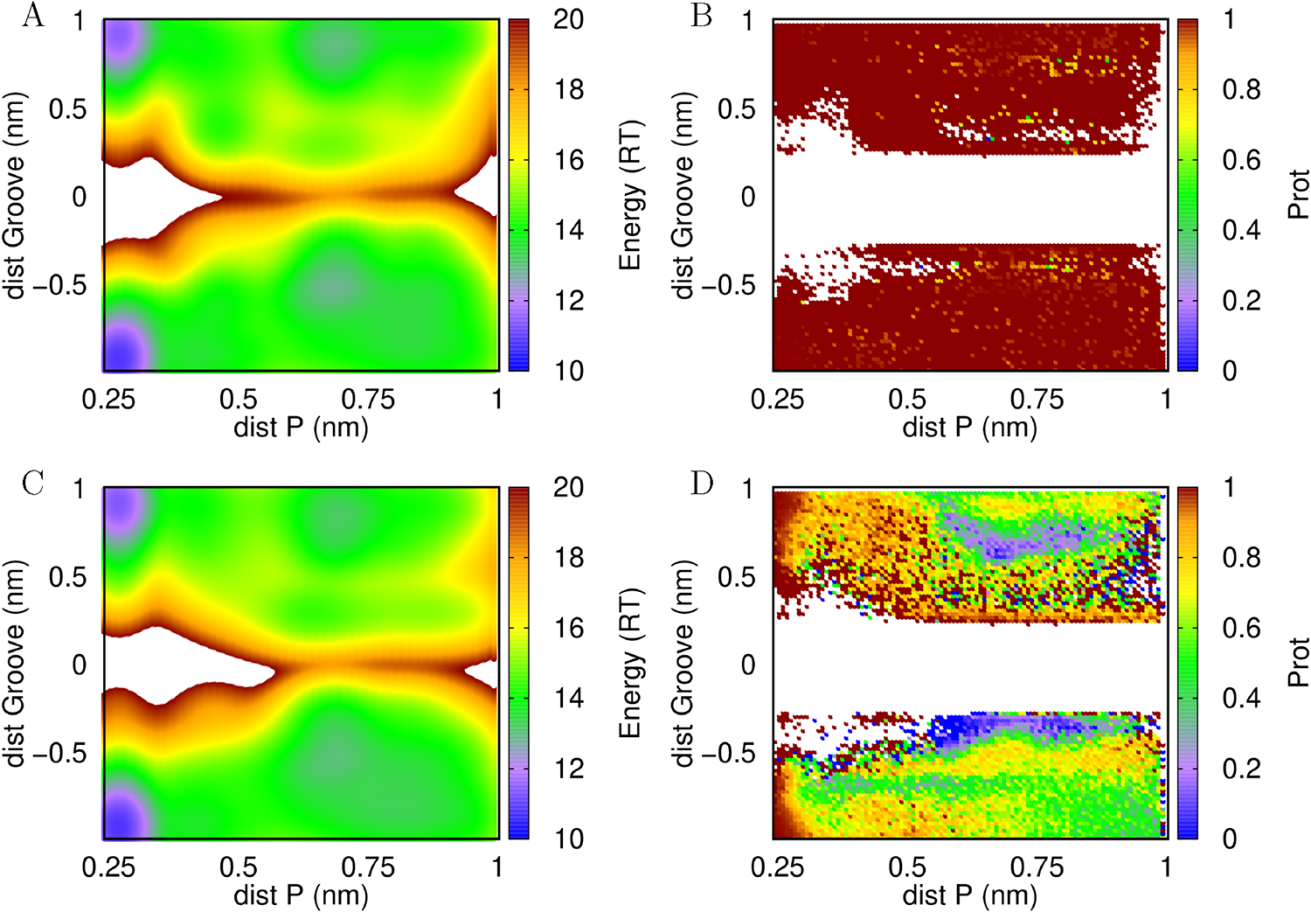
Energy landscapes of dendrimer N-termini average distance to closest siRNA groove vs dendrimer distance to the closest phosphate group (A, C) and their protonation scatterplots (B, D) at pH 5.0 (A, B) and 7.0 (C, D). The positive and negative groove distances refer to the positions where the amino group is closer to either the major or minor grooves, respectively. Figure S6 in the Supporting Information also provides a convergence assessment of these energy landscapes.

The energy landscapes show that all amino groups exhibit a clear preference for phosphate interactions, with a strong blue band (indicating low energy/high probability) at distances below 0.3 nm. This is true for all cases, even for the N-termini at pH 7, since the phosphate negative environment induces almost complete protonation of these residues also at this pH (Figure 5D). Indeed, while lysine side chains deprotonate only slightly, regardless of pH, the N-termini exhibit a rather complex protonation profile at pH 7. At this pH, these groups tend to deprotonate more when in the vicinity (within ∼0.4 nm) of the major and minor grooves, and tend to protonate more when interacting with the phosphate groups. This phenomenon was observed for all dendrimers and can be attributed to the proximity or shielding by the highly negatively charged region, which promotes protonation and deprotonation, respectively. The deprotonation of the N-termini is clearly more pronounced when the interaction is with the minor groove (negative distances in the plots), indicating that this shallower groove provides a slightly less negatively charged electrostatic potential and helps stabilize the neutral amino groups (Figure 6).

**Figure 6:**
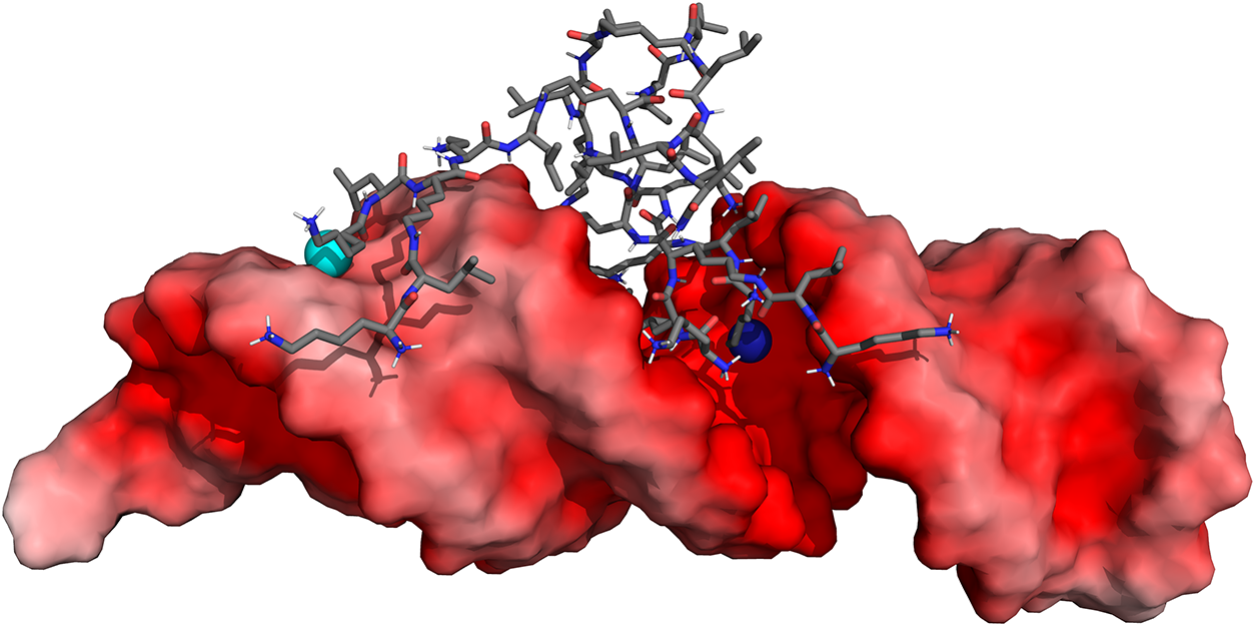
Structural representation of the MH18:siRNA complex, highlighting the interactions of the dendrimer N-termini with either the minor or the major grooves of siRNA. The red surface on the nucleic acid represents the negative electrostatic potential, while the dendrimer is depicted by gray sticks, with nitrogen and oxygen atoms colored blue and red, respectively. The light- and dark-blue spheres represent amine groups interacting with the minor and major grooves, respectively.

The differences between the configurational landscapes of all four dendrimers are very subtle (Table S1 of the Supporting Information), suggesting that their binding affinities to siRNA may also be similar. To investigate this, we employed two methods to estimate the binding affinities from our CpHMD simulations: pH-weighted MM/PBSA^46,47^ and ΔΔ*G* calculations using the Wyman–Tanford linkage (WT-Link) model (Table 3 and Tables S2–S3 of Supporting Information).^47,51–53^

**Table 3:** Binding Free Energies (in kcal/mol) Calculated Using the MM/PBSA and the Wyman–Tanford Linkage (WT-Link) Approaches^a^

	MMPBSA			
	pH 5.0	pH 7.0	ΔΔ*G* ^MMPBSA^	ΔΔ*G* ^WT–Link^
MH18	–34.9 ± 0.8	–30.3 ± 0.8	–4.6 ± 0.8	–4.4 ± 0.1
MH18D3	–35.5 ± 1.3	–32.5 ± 2.6	–3.0 ± 1.9	–3.4 ± 0.6
DMH18	–33.3 ± 1.4	–32.0 ± 1.6	–1.3 ± 1.5	–3.6 ± 0.2
MH47	–30.0 ± 1.1	–27.9 ± 1.4	–2.1 ± 1.2	–2.7 ± 0.6

a Since the linkage model only provides an energy difference between pH values, we calculated the ΔΔG^pH5–pH7^ values for both methods. The MM/PBSA free energies time series are also available in Figure S7 of the Supporting Information. The error values were calculated from the standard error of the mean using the replicate simulations.

MM/PBSA calculations are known to yield unreliable absolute binding energy values, with quite large error bars. However, when our objective is to rank a set of structurally similar binding complexes, this approximation provides a reasonable strategy at a moderate computational cost.^54^ The decomposition of the MM/PBSA binding energies shows that the polar terms (*E*_*Coul*_ and *Solv*_*polar*_) are highly prevalent, confirming the electrostatic nature of the dendrimer:siRNA interactions. The pH effects are also evident in the binding affinities, which tend to increase with acidity. This is intuitive, as a decrease in pH, as previously observed, results in the dendrimer becoming more charged and protonated, thereby enhancing its binding affinity for the oppositely charged siRNA. The method also identified MH47 as the weaker binder, as expected, since it is less charged at both pH values. For comparison with the WT-Link data (ΔΔ*G* ^WT–Link^), we calculated the corresponding ΔΔ*G* ^MMPBSA^ values, which show good overall agreement, except for DMH18. It should be noted that, unlike the MM/PBSA, the WT-Link approach is exact, without approximations, and the ΔΔ*G* values obtained are a direct result of the increase in protonation that occurs when pH is lowered from 7.0 to 5.0. The N-termini primarily drive this energy difference, as these groups undergo the most significant protonation changes between the two pH values. Therefore, the lower ΔΔ*G* ^WT–Link^ value observed for MH47 is not due to its reduced overall charge, but rather to the fact that its N-termini titrate within a less optimal pH range, leading to a smaller protonation difference between pH 7.0 and 5.0 compared to the other dendrimers.

Based on the free siRNA experimental data, MH18D3 should be the worst binder to siRNA, while MH47 should be the best (Table 1). The apparent mismatch between our computational binding free energies and the observed percentage of free siRNA prompts us to consider the possibility that the percentage of free siRNA may not directly correlate with a single dendrimer’s affinity for siRNA. Given that the dendrimer carries the siRNA molecules in the form of an assembled nanoparticle,^16^ and it is in this form that the dendrimer-siRNA complex is internalized in the cell via endocytosis, hence, the propensity of the dendrimer to form higher-order assemblies, with more dendrimer units, can also influence this factor. Indeed, MH47 is the more hydrophobic dendrimer (Figure S4 of Supporting Information), and tends to form larger aggregates (>4; limited solubility and tendency to precipitate), while MH18 and DMH18 tend to form aggregates with ∼4 dendrimers, and MH18D3 forms aggregates with only ∼2 dendrimers.^16^ From the data, we observe that the binding affinity of individual monomers is surpassed by that of higher-order aggregates, which exploit their increased multivalency to bind siRNA molecules more efficiently. This could help explain why MH47 is a weaker binder in its monomeric form, yet, in larger aggregates, it appears to sequester siRNA from solution more efficiently.

Despite these promising results and the model’s ability to explain most of the experimental observations, it is also important to note that some details remain elusive. Regarding chirality, our computational models did not show significant differences between l- and d-amino acid dendrimers. This suggests that the experimental data, in particular the aggregation propensities, depend on small, subtle stereospecific effects on the overall conformational landscape that our computational model does not capture. Another limitation is the inability to provide information about dendrimer aggregates and their interactions with siRNA. We interpreted the experimental data using approximate aggregation numbers (estimated from hydrodynamic radii) and by extrapolating the behavior of the dendrimer monomer to higher-order assemblies, which may not be entirely accurate, as each dendrimer might aggregate differently. However, simulating such large aggregates would pose serious problems, including sampling limitations and concentration effects.

## Conclusions

In this study, we analyzed the configurational and conformational landscapes of different peptide dendrimers interacting with a siRNA molecule. We assessed the roles of chirality, charge density, and pH in this interaction. We observed that the siRNA structure is not significantly affected by the dendrimer. However, the dendrimer’s amino groups predominantly interact with the negatively charged phosphate groups, inducing greater spread and charged conformations. These interactions are further enhanced at low pH, due to increased protonation of the dendrimer amines. Furthermore, this interaction is primarily modulated by the dendrimer’s N-termini, as these groups titrate and alter the dendrimer’s charge at physiological pH values (5.0–7.0). Despite experimental evidence showing that the more hydrophobic dendrimer, MH47, exhibits the best affinity for siRNA, we found the opposite to be true. We showed that fewer Lys residues result in a weaker binder due to a smaller overall charge. The experimental and computational data are reconciled by introducing the aggregation propensities of the different dendrimers, which are not captured in our CpHMD simulations. These are very high for MH47 and help explain its ability to better sequester siRNA from solution than the other dendrimer sequences. A similar interpretation could explain why the chiral MH18D3 shows weaker performance, as this dendrimer has the lowest aggregation propensity.^16^ Our results suggest that chirality has only a small impact on the dendrimer/siRNA binding modes, and these differences are difficult to capture, especially at the monomer level. Overall, this work provides key insights into the interaction between a dendrimer monomer and an siRNA molecule, as well as potential strategies to modulate this interaction, which can be used to design more efficient dendrimers.

## Supplementary Material





## Data Availability

The GROMACS package is freely available software for MD simulations and can be downloaded at https://manual.gromacs.org/documentation/2020.1/download.html. PyMOL v3.1 is also free software for molecular visualization and high-quality image generation. It can be downloaded from https://pymol.org. A zip file with all topologies, system configurations, parameter files, and the CpHMD code is also provided.

## Notes

Time series with the dendrimer radius of gyration, total charge, siRNA heavy atoms RMSD, siRNA bending, dendrimer position along the siRNA length vector, interfacial area, number of phosphate-amine contacts, electrostatic shielding, and dendrimer:siRNA binding energies at different pH values. Percentage of the total hydrophobic interfacial area and dendrimer hydrophobic area exposed to water. Energy landscapes of dendrimer amine distance to siRNA grooves versus phosphate groups, and their protonation scatterplots. Binding free energy decompositions using both MM/PBSA and Wyman–Tanford linkage methods (PDF)
A zip file containing all topologies, system configurations, and parameter files for the CpHMD code is also provided (ZIP)
