# Supplementary material for: Constant-pH Molecular Dynamics of Cationic Peptide Dendrimers Binding to siRNA
Source: J Chem Inf Model. 2026 Feb 5;66(8):4734–43. doi: 10.1021/acs.jcim.5c02636 (PMC13126635; doi:10.1021/acs.jcim.5c02636)
Supplement: Supplementary file 1 [file ci5c02636_si_001.pdf]

Supporting Information: Constant-pH Molecular Dynamics of Cationic Peptide Dendrimers binding to siRNA

Filipe E. P. Rodrigues,[†] Tamis Darbre,[‡] and Miguel Machuqueiro[*,†]

[†]*BioISI – Instituto de Biossistemas e Ciências Integrativas, Departamento de Química e Bioquímica, Faculdade de Ciências, Universidade de Lisboa, 1749-016, Lisboa, Portugal*

[‡]*Department of Chemistry, Biochemistry and Pharmaceutical Sciences, University of Bern, Bern, Switzerland*

E-mail: machuque@ciencias.ulisboa.pt

Phone: +351-21-7500112

List of Tables

S1 Energy landscapes of dendrimer amines average distance to closest siRNA groove vs dendrimer distance to the closest phosphate group S3
S2 Binding free energies calculated using PyBindE S4
S3 Binding free energies calculated using the Wyman–Tanford linkage approach S4

List of Figures

S1 Dendrimer radius of gyration, total charge, siRNA heavy atoms RMSD and bending for all dendrimers S5
S2 Dendrimer position along the siRNA length vector, interfacial area, number of phosphate-amine contacts, and electrostatic shielding, for all dendrimers . S6
S3 Dendrimer-siRNA interface area, and electrostatic shielding convergence . . . S7
S4 Percentage of the total hydrophobic interfacial area for all dendrimers S7
S5 Dendrimer hydrophobic area exposed to water S8
S6 Energy landscapes of dendrimer N-termini average distance to closest siRNA groove vs dendrimer distance to the closest phosphate group (A,C,E,G) and their protonation scatterplots (B,D,F,H) at pH 5.0 (A,B,C,D) and 7.0 (E,F,G,H). The plots were computed using the first half of our sampling (A,B,E,F) to compare with the second half (C,D,G,H). The positive and negative groove distances refer to the positions where the amino group is closer to either the major or minor grooves, respectively. S9
S7 Dendrimer binding energy, and its energetic contributions, namely: Coulombic, Van der Waals, polar, and apolar S10

Table S1: Energy landscapes of dendrimer amines average distance to closest siRNA groove vs dendrimer distance to the closest phosphate group (abundance) and their protonation scatterplots (prot) at pH 5 and 7. Each distinct group of amines is represented separately, namely the N-termini and the lysine side chains of 2nd and 3rd generations. The positive and negative groove distances refer to the positions where the amino group is closer to either the major or minor grooves, respectively.

		G3-NTR		G3-side		G2	
		abundance	prot	abundance	prot	abundance	prot
MH18	pH 5	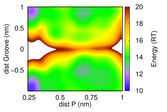	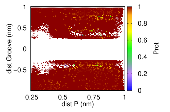	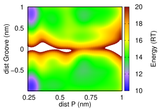	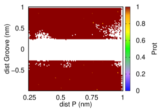	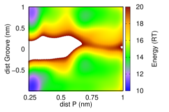	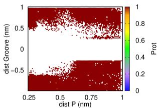
	pH 7	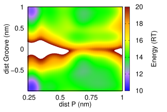	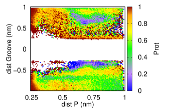	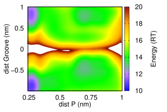	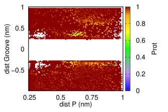	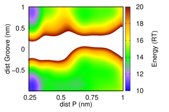	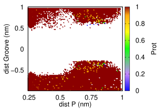
MH18D3	pH 5	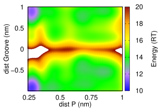	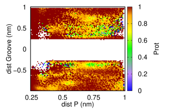	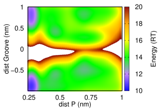	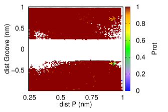	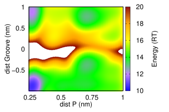	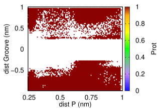
	pH 7	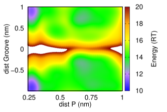	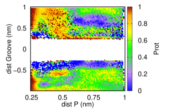	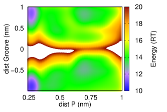	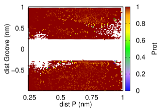	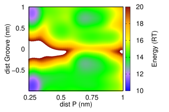	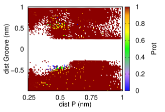
DMH18	pH 5	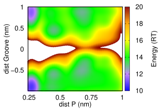	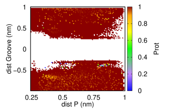	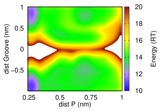	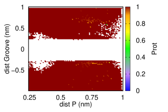	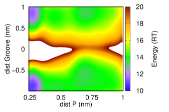	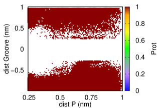
	pH 7	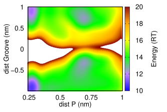	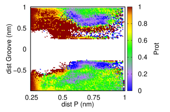	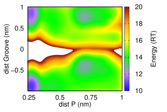	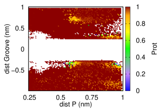	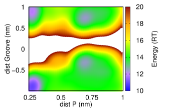	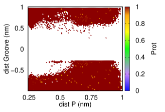
MH47	pH 5	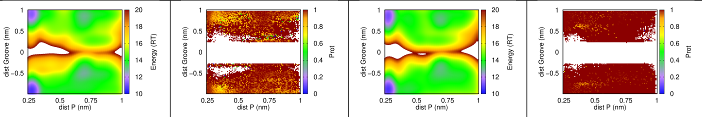				-	-
	pH 7	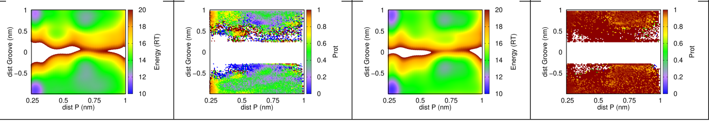				-	-

Table S2: Binding free energies (kcal/mol) calculated using PyBindE. We also present the decomposition of the free energy into the different terms: the MM energies in vacuum, namely Van der Waals (E_{VdW}) and Coulombic (E_{Coul}) terms, and two related to the solvation energy, polar ($Solv_{polar}$) and apolar ($Solv_{apolar}$).

		MH18	MH18D3	DMH18	MH47
E_{bind}	pH 5	-34.9 ± 0.8	-35.5 ± 1.3	-33.3 ± 1.4	-30.0 ± 1.1
	pH 7	-30.3 ± 0.8	-32.5 ± 2.6	-32.0 ± 1.6	-27.9 ± 1.4
$\Delta\Delta G^{pH5-pH7}$		-4.6	-3.0	-1.3	-2.1
E_{Coul}	pH 5	-3117.5 ± 80.0	-3237.7 ± 61.1	-3100.3 ± 82.1	-2596.2 ± 45.0
	pH 7	-3178.4 ± 59.3	-3228.2 ± 86.7	-3280.6 ± 82.2	-2504.8 ± 55.0
E_{VdW}	pH 5	-30.8 ± 1.0	-29.7 ± 1.9	-28.9 ± 2.8	-27.8 ± 2.5
	pH 7	-31.1 ± 1.5	-34.3 ± 3.0	-32.7 ± 2.9	-31.3 ± 1.5
$Solv_{apolar}$	pH 5	-9.7 ± 0.3	-9.7 ± 0.4	-9.1 ± 0.5	-8.8 ± 0.5
	pH 7	-10.0 ± 0.3	-10.4 ± 0.6	-9.9 ± 0.6	-8.9 ± 0.3
$Solv_{polar}$	pH 5	3123.2 ± 80.3	3241.6 ± 60.5	3105.0 ± 81.6	2602.8 ± 45.7
	pH 7	3189.1 ± 59.8	3240.4 ± 87.9	3291.1 ± 84.0	2517.0 ± 56.1

Table S3: Binding free energies (kcal/mol) calculated using the Wyman–Tanford linkage approach. Since the model only provides ΔΔG values between pH values, we present here the energy difference between pH 5 and 7 ($\Delta\Delta G^{pH5-pH7}$).

	MH18	MH18D3	DMH18	MH47
Full	-4.4 ± 0.1	-3.4 ± 0.6	-3.6 ± 0.2	-2.7 ± 0.6
G2	0.0 ± 0.0	0.0 ± 0.0	0.0 ± 0.0	—
G3-side	-0.1 ± 0.0	-0.1 ± 0.0	-0.1 ± 0.0	0.0 ± 0.0
G3-Ntr	-4.4 ± 0.1	-3.3 ± 0.5	-3.5 ± 0.2	-2.7 ± 0.6

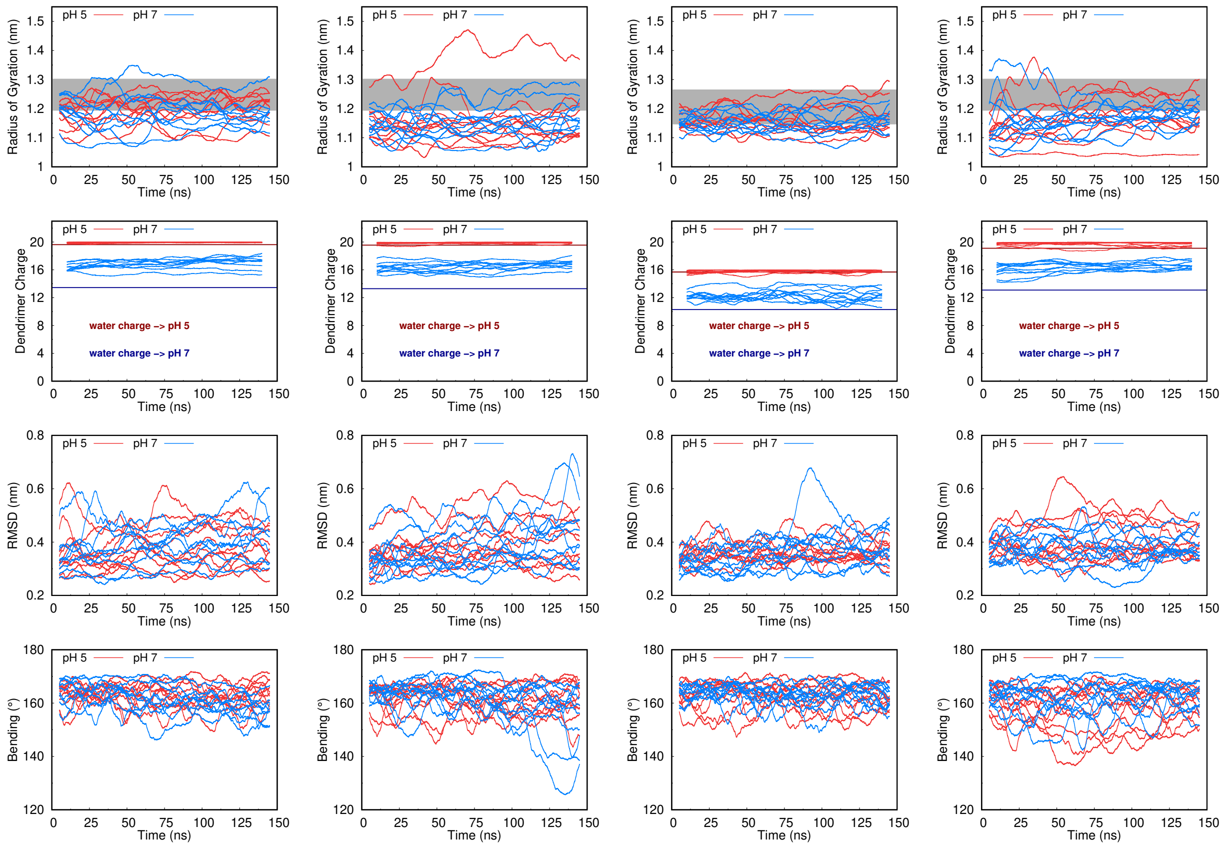

Figure S1: Dendrimer radius of gyration (1st row), total charge (2nd row), siRNA heavy atoms RMSD (3rd row) and bending (4th row) for MH18 (1st column), DMH18 (2nd column), MH47 (3rd column), and MH18D3 (4th column). Replicates at pH 5 and 7 are represented in red and blue, respectively. For the dendrimer radius of gyration plots, a gray area is shown to represent the range of the radius of gyration that is sampled by dendrimers at pH 4–8 in the water simulations. For DMH18, the radius of the gyration range of MH18 was used. A sliding-window average over 10 ns was applied to remove undesired fast fluctuations.

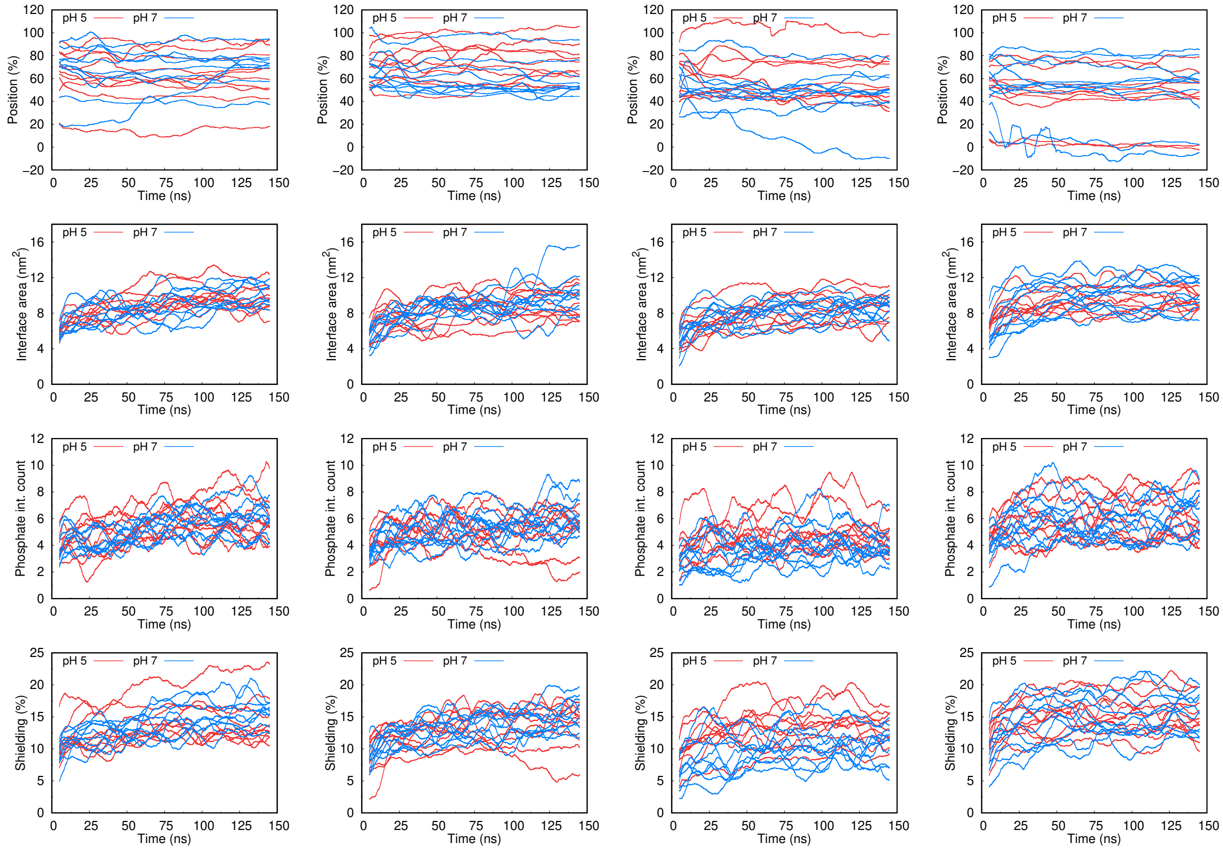

Figure S2: Dendrimer position along the siRNA length vector (1st row), interfacial area (2nd row), number of phosphate-amine contacts (3rd row), and electrostatic shielding (4th row), for MH18 (1st column), DMH18 (2nd column), MH47 (3rd column) and MH18D3 (4th column). Replicates at pH 5 and 7 are represented in red and blue, respectively. A sliding-window average over 10 ns was applied to remove undesired fast fluctuations.

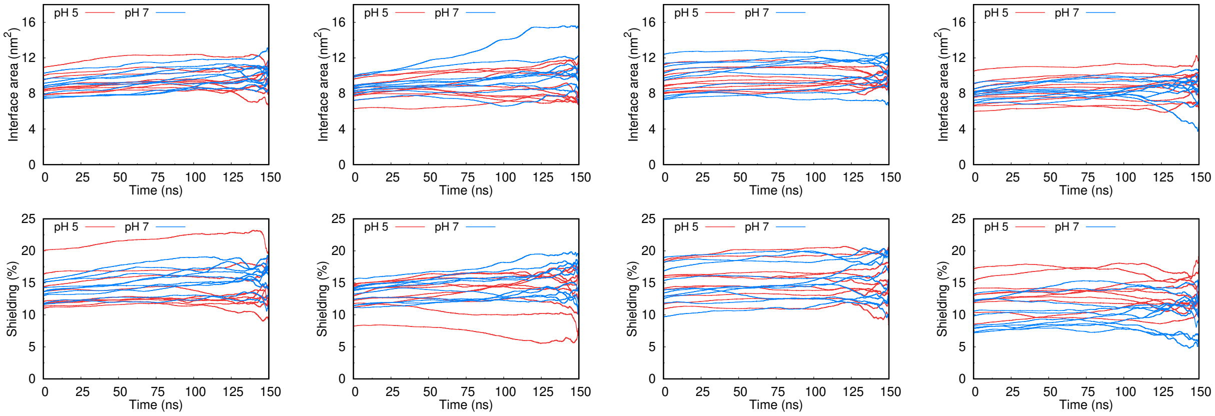

Figure S3: Dendrimer-siRNA complex interface area (1st row), and electrostatic shielding (2nd row) cumulative averages over time. These were calculated starting from the end of the simulation. The results are shown for MH18 (1st column), DMH18 (2nd column), MH18D3 (3rd column) and MH47 (4th column). Replicates at pH 5.0 and 7.0 are represented in red and blue, respectively.

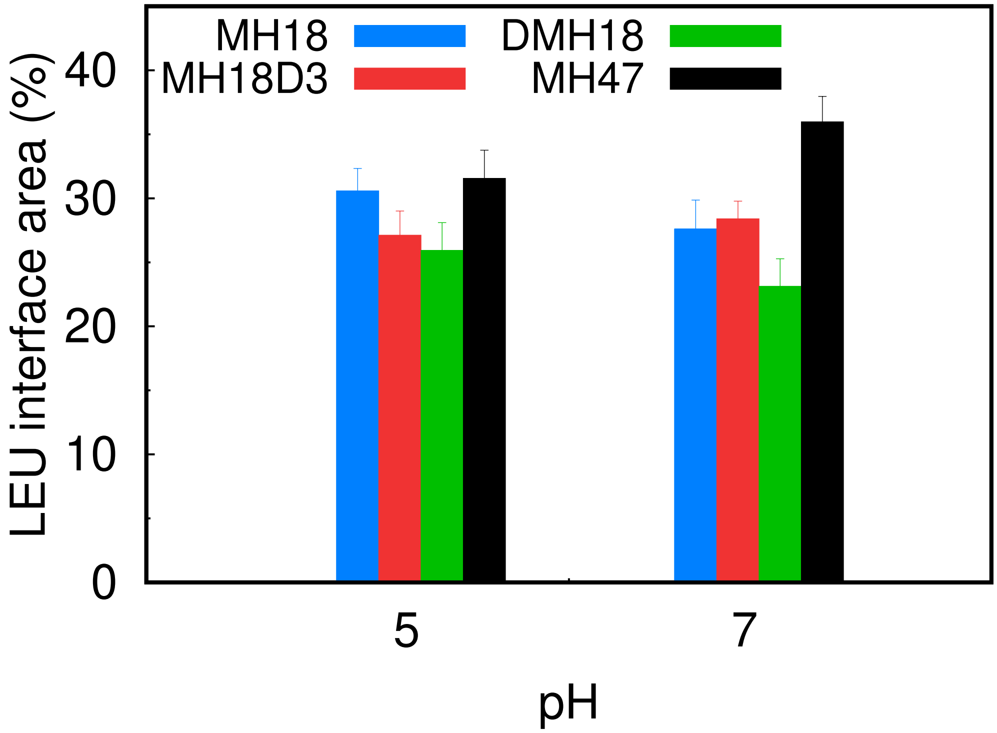

Figure S4: Percentage of the total hydrophobic interfacial area for all dendrimer systems at pH 5 and 7.

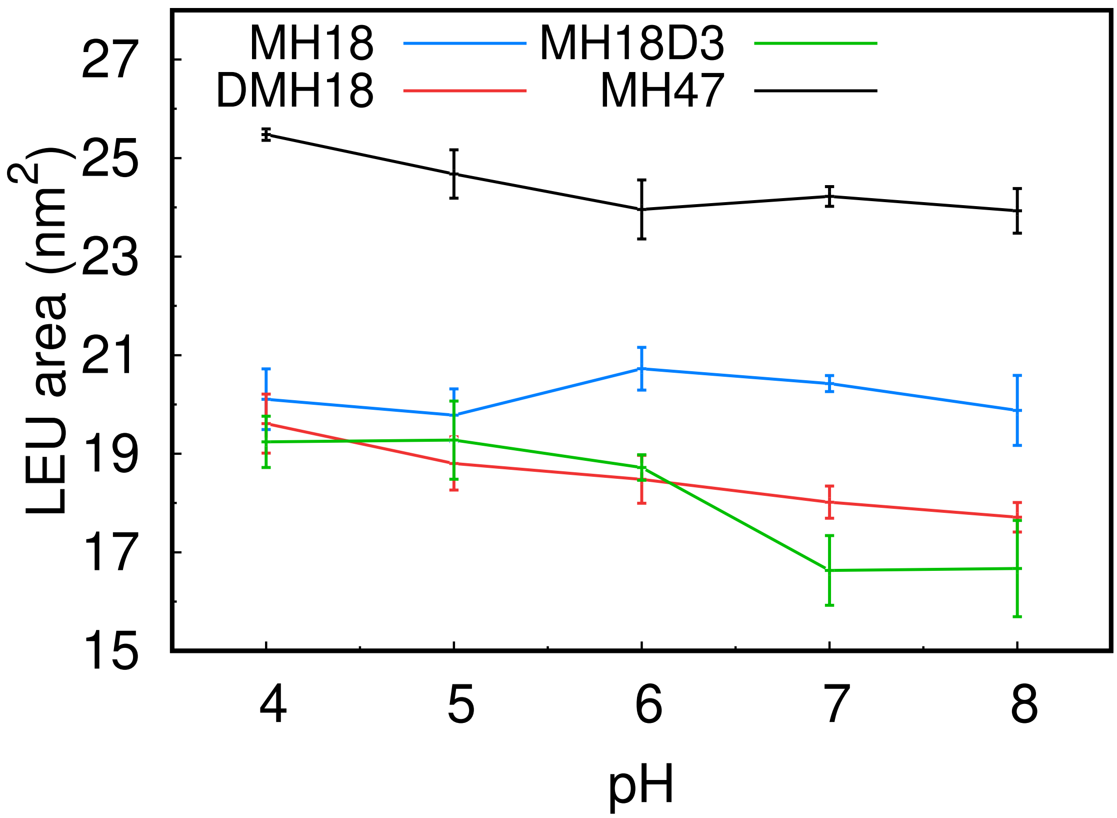

Figure S5: Dendrimer hydrophobic (Leu) area exposed to water, over pH. The data were obtained from the dendrimer simulations without the nucleic acid.

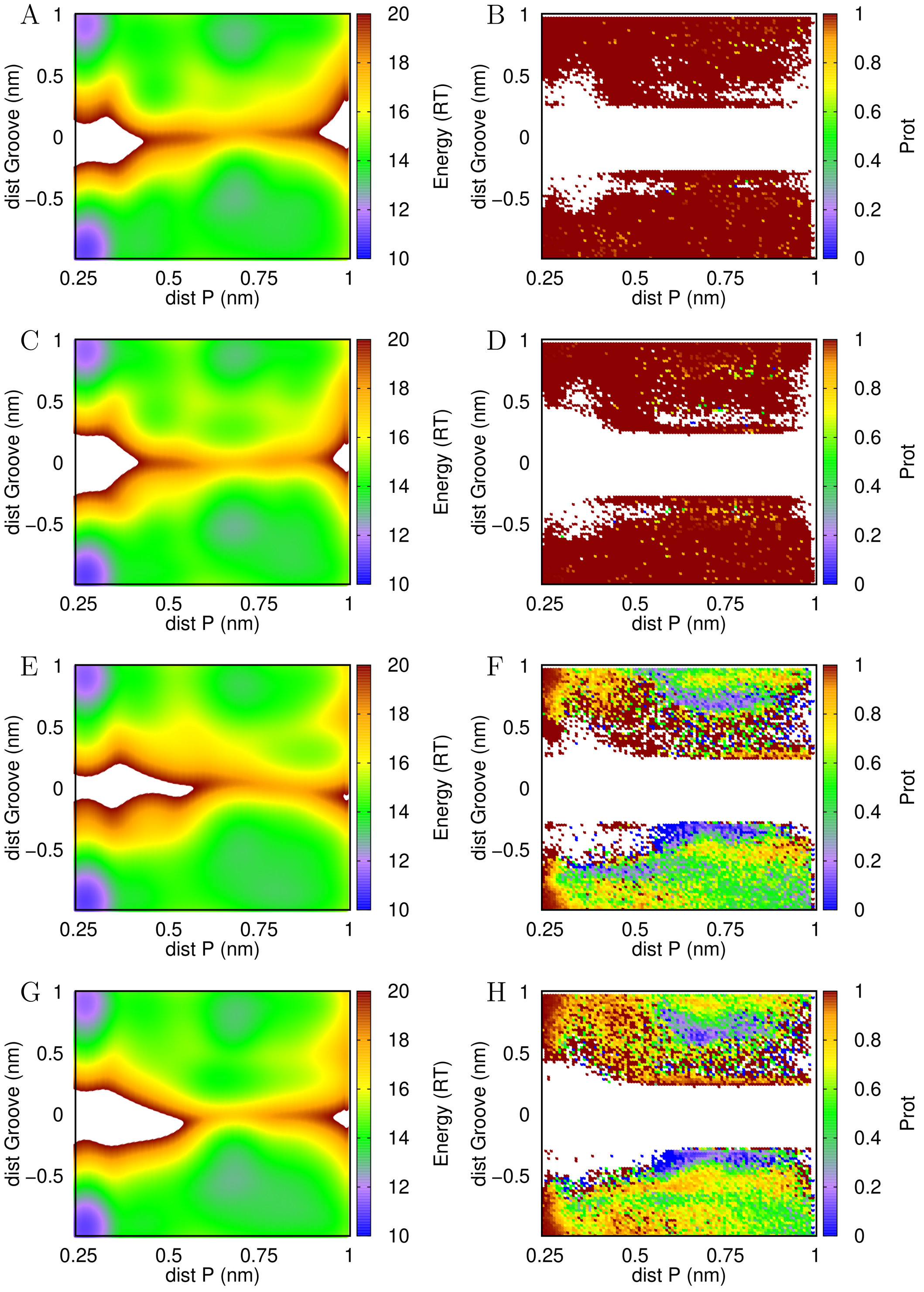

Figure S6: Energy landscapes of dendrimer N-termini average distance to closest siRNA groove vs dendrimer distance to the closest phosphate group (A,C,E,G) and their protonation scatterplots (B,D,F,H) at pH 5.0 (A,B,C,D) and 7.0 (E,F,G,H). The plots were computed using the first half of our sampling (A,B,E,F) to compare with the second half (C,D,G,H). The positive and negative groove distances refer to the positions where the amino group is closer to either the major or minor grooves, respectively.

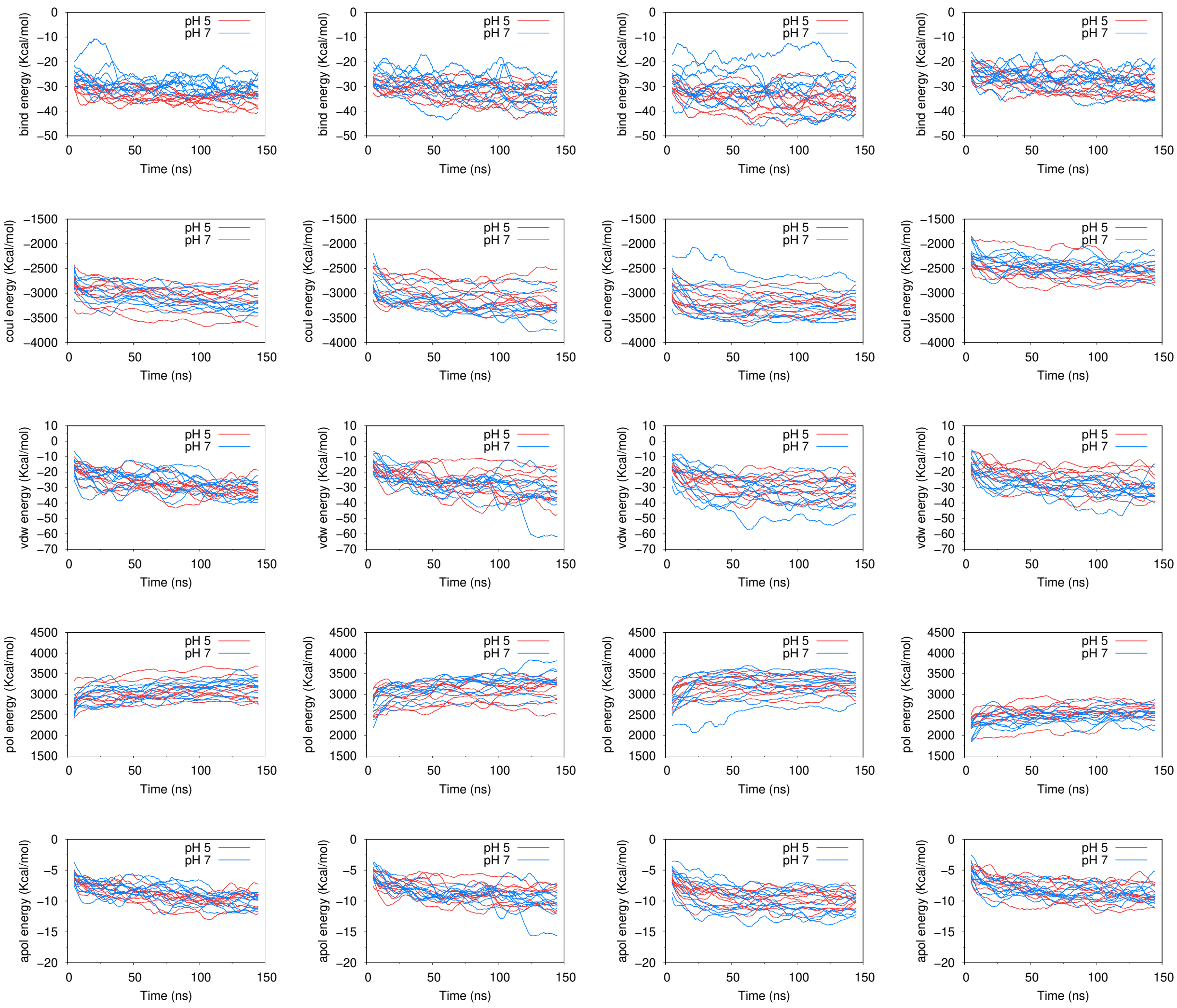

Figure S7: Dendrimer binding energy (1st row), and its energetic contributions, namely: Coulombic (2nd row), Van der Waals (3rd row), polar (4th row), and apolar (5th row). The results are shown for MH18 (1st column), DMH18 (2nd column), MH18D3 (3rd column) and MH47 (4th column). Replicates at pH 5.0 and 7.0 are represented in red and blue, respectively. A sliding-window average over 10 ns was applied to remove undesired fast fluctuations.
